# Complicated hospitalization due to influenza: results from the Global Hospital Influenza Network for the 2017–2018 season

**DOI:** 10.1186/s12879-020-05167-4

**Published:** 2020-07-02

**Authors:** Bruno Lina, Alexandre Georges, Elena Burtseva, Marta C. Nunes, Melissa K. Andrew, Shelly A. McNeil, Guillermo M. Ruiz-Palacios, Luzhao Feng, Jan Kyncl, Philippe Vanhems, Justin R. Ortiz, John Paget, Robert C. Reiner, on behalf of the GIHSN 2017–2018 study collaborators, Sélilah Amour, Coulibaly Anderson N’Gattia, Victor Baselga Moreno, Elsa Baumeister, Jalila Ben Khelil, Daria Danilenko, Javier Diez-Domingo, Anca Cristina Drăgănescu, Gideon O. Emukule, Zhetpisbayeva Gauhar, M. Lourdes Guerrero, Ainara Mira-Iglesias, Lidija Kisteneva, Parvaiz A. Koul, Ainagul Kuatbaeva, Victor Alberto Laguna Torres, Odile Launay, Nezha Lenzi, Shabir Madhi, Zdenka Mandakova, Snežana Medić, Mioljub Ristić, Hyder Mir, Aneta Nitsch-Osuch, Nancy Otieno, Daniela Pițigoi, Andrea Pontoriero, Estela Ramirez, Ben Salah, Oana Sandulescu, Natali Serafin, Wei Shan, Anna Sominina, Svetlana Trushakova, Andrzej Zalewski, Tao Zhang

**Affiliations:** 1 CIRI, Lyon University, Inserm U 1111, Lyon, France; 2 Hospices Civils de Lyon, Croix-Rousse University Hospital, Infectious Agents Institute (IAI) Laboratory of Virology-National Reference Center for Respiratory Viruses (Including Influenza), Lyon, France; 3 Claude Bernard University (Lyon 1), Lyon, France; 4 OpenHealth, Paris, France; 5 FSBI “N.F. Gamaleya NRCEM”, Moscow, Russian Federation; 6 Medical Research Council: Respiratory and Meningeal Pathogens Research Unit, School of Pathology, Faculty of Health Sciences, University of the Witwatersrand, Johannesburg, South Africa; 7 Department of Science and Technology/National Research Foundation: Vaccine Preventable Diseases Unit, University of the Witwatersrand, Johannesburg, South Africa; 8 Canadian Center for Vaccinology, IWK Health Centre and Nova Scotia Health Authority, Dalhousie University, Halifax, Canada; 9 National Institutes of Health, Mexico City, Mexico; 10 Division of Infectious Diseases, Chinese Center for Disease Control and Prevention, Beijing, China; 11 National Institute of Public Health, Prague, Czech Republic; 12 Groupement Hospitalier Edouard Herriot, Unité d’Hygiène, Epidémiologie et Prévention, Hospices Civils de Lyon, Lyon, France; 13 Emerging Pathogens Laboratory – Epidemiology and International Health, Fondation Mérieux, Centre International de Recherche en Infectiologie (CIRI), Lyon, France; 14 Inserm, F-CRIN, Innovative Clinical Research Network in Vaccinology (I-REIVAC), CIC, 1417 Paris, France; 15 Center for Vaccine Development and Global Health, University of Maryland School of Medicine, Baltimore, MD USA; 16 Netherlands Institute for Health Services Research (NIVEL), Utrecht, The Netherlands; 17 Institute of Health Metrics and Evaluation, Department of Health Metrics Sciences, University of Washington, Seattle, WA USA

**Keywords:** Influenza, Hospitalization, Mortality, Risk factors, Epidemiology

## Abstract

**Background:**

Since 2011, the Global Influenza Hospital Surveillance Network (GIHSN) has used active surveillance to prospectively collect epidemiological and virological data on patients hospitalized with influenza virus infection. Here, we describe influenza virus strain circulation in the GIHSN participant countries during 2017–2018 season and examine factors associated with complicated hospitalization among patients admitted with laboratory-confirmed influenza illness.

**Methods:**

The study enrolled patients who were hospitalized in a GIHSN hospital in the previous 48 h with acute respiratory symptoms and who had symptoms consistent with influenza within the 7 days before admission. Enrolled patients were tested by reverse transcription-polymerase chain reaction to confirm influenza virus infection. “Complicated hospitalization” was defined as a need for mechanical ventilation, admission to an intensive care unit, or in-hospital death. In each of four age strata (< 15, 15–< 50, 50–< 65, and ≥ 65 years), factors associated with complicated hospitalization in influenza-positive patients were identified by mixed effects logistic regression and those associated with length of hospital stay using a linear mixed-effects regression model.

**Results:**

The study included 12,803 hospitalized patients at 14 coordinating sites in 13 countries, of which 4306 (34%) tested positive for influenza. Influenza viruses B/Yamagata, A/H3N2, and A/H1N1pdm09 strains dominated and cocirculated, although the dominant strains varied between sites. Complicated hospitalization occurred in 10.6% of influenza-positive patients. Factors associated with complicated hospitalization in influenza-positive patients included chronic obstructive pulmonary disease (15–< 50 years and ≥ 65 years), diabetes (15–< 50 years), male sex (50–< 65 years), hospitalization during the last 12 months (50–< 65 years), and current smoking (≥65 years). Chronic obstructive pulmonary disease (50–< 65 years), other chronic conditions (15–< 50 years), influenza A (50–< 65 years), and hospitalization during the last 12 months (< 15 years) were associated with a longer hospital stay. The proportion of patients with complicated influenza did not differ between influenza A and B.

**Conclusions:**

Complicated hospitalizations occurred in over 10% of patients hospitalized with influenza virus infection. Factors commonly associated with complicated or longer hospitalization differed by age group but commonly included chronic obstructive pulmonary disease, diabetes, and hospitalization during the last 12 months.

## Background

Each year, seasonal influenza epidemics cause an estimated 3 to 5 million severe illnesses and 290,000 to 650,000 deaths worldwide [1, 2]. Young children, older adults, pregnant women, immunocompromised individuals, and patients of any age with cardiopulmonary conditions or other chronic diseases are considered to have the highest risk of severe influenza illness [3, 4]. Hospitalization and death due to influenza, however, can also occur in individuals who were previously healthy [5].

Several countries collect substantial data on severe cases of influenza. In the United States, for example, the Centers for Disease Control and Prevention collect data on hospitalizations with laboratory-confirmed influenza illness through a network covering approximately 9% of the US population [6]. These data are used to make decisions about US prevention strategies, diagnosis, and treatment. However, more detailed data are needed from geographically diverse settings over several influenza seasons to assess how the different influenza viruses affect clinically meaningful outcomes. Recognizing this need, the Global Influenza Hospital Surveillance Network (GIHSN) was established in 2011 [7]. The network includes geographically dispersed and diverse sites linked with local health authorities, each of which coordinates influenza surveillance at participating hospitals according to a common core protocol. Influenza virus infection is confirmed by reverse transcription-polymerase chain reaction (RT-PCR), and to facilitate pooling between sites, the network takes several steps to improve data quality and comparability. Data collected by the GIHSN provide an opportunity to investigate the associations between severe illness, patient characteristics, and influenza virology. In the 2017–2018 season, the GIHSN included 20 coordinating sites in 19 countries on five continents.

The GIHSN has reported epidemiological findings from 2012–2013 [5], 2013–2014 [8], 2014–2015 [9], and 2016–2017 influenza seasons [10]. The studies have shown that all strains of influenza can result in hospitalization, intensive care unit (ICU) admission, or death and that the strains responsible for severe cases vary substantially between seasons, sites, and even within single regions or countries. In addition, these studies have confirmed that influenza can cause serious outcomes not only in older adults, individuals with comorbidities, and pregnant women but also in the wider population, irrespective of age, sex, or comorbidities [5, 8, 9].

Herein we report the results from the GIHSN for the 2017–2018 influenza season. In addition to describing the epidemiology of hospitalized cases of influenza, we focused on identifying factors associated with complicated hospitalization in these patients.

## Methods

### Overall methodology of the GIHSN

The GIHSN uses prospective active surveillance to collect epidemiological and virological data from patients hospitalized with acute respiratory symptoms during the influenza season [11]. All sites share the same core protocol and use RT-PCR to confirm influenza virus infection. The core protocol was approved by the institutional review board or ethics committee of each participating site.

To be eligible, patients had to reside in the predefined catchment area of a participating hospital, be hospitalized in the previous 48 h with acute respiratory symptoms, and not live in an institutionalized setting. Healthcare professionals trained to follow the GIHSN study protocol approached eligible patients. Patients aged > 5 years had to have at least one systemic symptom (fever/feverishness, malaise, headache, or myalgia) and at least one respiratory symptom (cough, sore throat, or shortness of breath) consistent with influenza and had to have been hospitalized ≤7 days after the onset of the symptoms (see Additional file 1 for diagnosis codes). Patients aged ≤5 years had to be hospitalized ≤7 days after the appearance of symptoms associated with influenza (see Additional file 2 for diagnosis codes). Patients were excluded if they had been discharged from a hospital < 30 days before the current episode.

After patients or their legal representatives provided informed consent, nasopharyngeal, nasal, oral, or oropharyngeal samples are obtained from each patient (see Additional file 3 for sample collection). Samples are placed in a single viral transport media tube and stored at ≤−20 °C at the study site or sent directly to the coordinating site’s reference laboratory for testing. Samples are collected within 48 h of hospital admission. Influenza virus infection is confirmed by RT-PCR, and positive samples are subtyped by RT-PCR to identify A(H1N1)pdm09, A(H3N2), B/Yamagata-lineage, and B/Victoria-lineage strains. Core questionnaires (one for patients aged < 5 years and one for patients aged ≥5 years), translated into the local language, are used to collect patient demographics, comorbidities, and influenza vaccination status through face-to-face interviews of patients or legal representatives, interviews of attending physicians, and a review of clinical records. Obesity is assessed only in adults (≥18 years). Direct exposure to smoking is assessed in participants ≥14 years of age and passive exposure to smoking in participants < 14 years of age. Obesity was defined as a body mass index > 30 kg/m^2^. Functional status is assessed by Barthel Index [12] in patients aged ≥65 years. Patients were considered vaccinated if they had received at least one dose of a 2017–2018 seasonal influenza vaccine ≥14 days before the onset of symptoms. Physicians involved in clinical care of patients may be involved in patient recruitment but are not involved in assessing eligibility for inclusion.

### Statistical analysis

Only patients with respiratory specimens collected within 7 days of symptom onset were included to reduce false negatives due to decreasing viral RNA levels over time [8]. Sites including fewer than 40 hospitalized patients were excluded from the current analysis to reduce variability. For each site, the inclusion period was defined by the weeks with positive specimens for influenza. Patients enrolled outside the inclusion period at each site were excluded from analysis. The co-primary endpoints were (a) the proportion of influenza-positive patients with “complicated hospitalization”, as defined by a need for mechanical ventilation support, admission to an ICU, or death during hospitalization; and (b) length of hospital stay in days. Age categories were as recommended by the World Health Organization for analysis of severe acute respiratory illness/influenza-like illness [13].

Strain circulation was analyzed by site and grouped by World Health Organization transmission zone [14]. The number of positive patients for each strain (A/H1N1pdm09, A/H3N2, A subtype unknown, B/Yamagata, B/Victoria, and B lineage unknown) was calculated separately; therefore, patients could have been infected with more than one strain.

Mixed effects logistic regression was used to identify factors associated with complicated hospitalization, with 95% confidence intervals (CIs) calculated by the Wald method. A linear mixed-effects regression model was used to identify factors associated with length of hospital stay (as a continuous variable), with 95% CIs calculated by profile-likelihood method. These analyses were conducted in four age strata (< 15, 15–< 50, 50–< 65, and ≥65 years). For admissions aged ≥15 years, covariates included cardiovascular disease, chronic obstructive pulmonary disease (COPD), diabetes, obesity, other chronic conditions, sex, current smoking, influenza type (A vs B), antiviral prescription during the current episode, hospitalization during the previous 12 months, site (as random effect), influenza vaccination status, and age. For admissions aged < 15 years, covariates included any chronic condition, sex, age, influenza type (A vs. B), antiviral prescription during the current episode, hospitalization during the previous 12 months, site (as random effect), influenza vaccination status, and age. Factors tested in the mixed effects logistic regression model were considered associated with complications if the 95% CI of the odds ratio (OR) did not cross 1. In the linear mixed-effects regression model, the coefficient indicated the change in length of hospital stay in days when the indicated factor was changed by one unit (i.e. from yes to no), and tested factors were considered associated with a longer hospital stay if the 95% CI of the coefficient did not cross 0.

Statistical analysis was performed using R software version 3.4.4 (R Core Team, 1993) or Excel version 1808 (Microsoft, Redmond, WA). Missing data were not replaced.

## Results

### Patients included

**Fig. 1:**
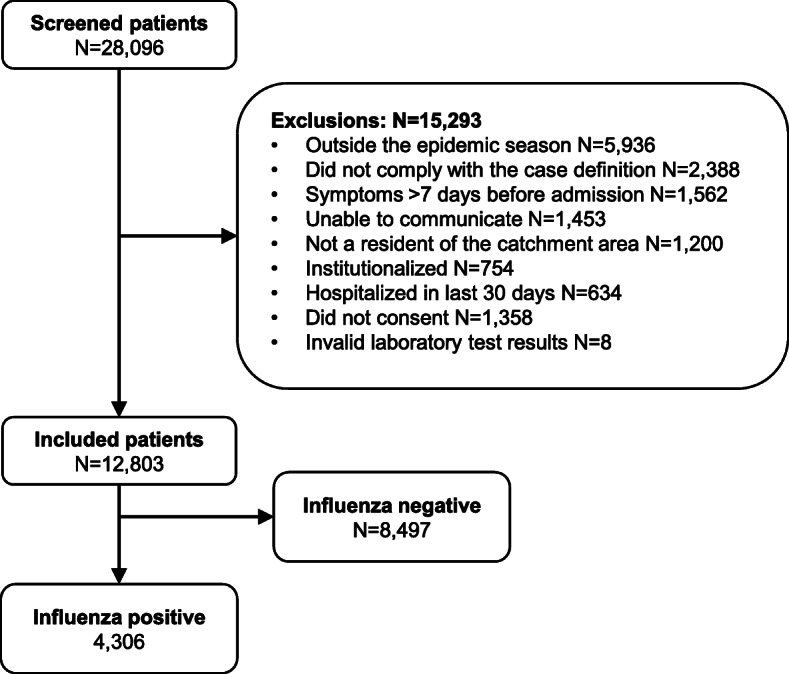
Disposition and influenza positivity of included patients. Community-dwelling patients residing in predefined catchment areas of the participating hospitals and hospitalized < 48 h with presenting complaints potentially associated with influenza were eligible. Patients aged > 5 years had to have ≥1 systemic symptom (fever/feverishness, malaise, headache, or myalgia) and ≥ 1 respiratory symptom (cough, sore throat, or shortness of breath) and had to have been hospitalized < 7 days of the onset of the symptoms. Patients aged ≤5 years had to be hospitalized < 7 days after the appearance of symptoms potentially associated with influenza. Patients were excluded if they had been discharged from a hospital < 30 days before the current episode. Influenza infection was confirmed and typed/subtyped by RT-PCR

A total of 28,096 hospitalized patients at 46 participating hospitals in 13 countries were screened for eligibility (Table 1). Six of the sites included in the GIHSN during the 2017–2018 influenza season (Paris [France], Ivory Coast, Kazakhstan, Peru, Poland, and Tunisia) were not included in the current analysis because they recruited fewer than 40 patients each. Of the 27,096 screened patients, 12,803 (47.3%) met selection criteria and were included in the analysis (Fig. 1). The most common reasons for exclusion were admission outside the epidemic season for the site, not having the required symptoms, or having had symptoms for > 7 days.

**Table 1:** Characteristics of included sites

Coordinating site	Number of hospitals	Included patients	Inclusion period (calendar week and year of influenza season)	Influenza transmission zone ^**a**^
St. Petersburg (Russian Federation)	7	3101	Week 2 to 21, 2018	Eastern Europe
Spain (Valencia)	4	2841	Week 45, 2017 to week 20, 2018	South West Europe
Moscow (Russian Federation)	1	1247	Week 3 to 19, 2018	Eastern Europe
South Africa (Johannesburg)	2	1123	Week 15 to 42, 2018	Southern Africa
Canada (Halifax)	14	1026	Week 50, 2017 to week 17, 2018	North America
Mexico (Mexico City)	6	701	Week 42, 2017 to week 13, 2018	Central America Caribbean
India (Srinagar)	1	609	Week 41, 2017 to week 9, 2018	Southern Asia
Serbia (Novi Sad)	4	590	Week 52, 2017 to week 15, 2018	South West Europe
Romania (Bucharest)	1	492	Week 49, 2017 to week 16, 2018	Eastern Europe
China (Shanghai)	1	399	Week 49, 2017 to week 11, 2018	Eastern Asia
Kenya (Nairobi)	2	386	Week 4 to 32, 2018	Eastern Africa
Czech Republic (Prague)	1	117	Week 52, 2017 to week 12, 2018	Eastern Europe
Lyon (France)	1	98	Week 50, 2017 to week 13, 2018	South West Europe
Argentina (Buenos Aires)	1	73	Week 30 to 39, 2018	Temperate South America
All sites	46	12,803		

^**a**^ World Health Organization influenza transmission zone [14]

Just over half (51.0%) of the 12,803 included patients were male (Table 2). Overall, 43.3% of the included patients were aged < 15 years and 26.4% were aged ≥65 years. Overall, more than half of the patients (58.6%) did not report any chronic conditions. Of patients with comorbidities or underlying diseases, the most common was cardiovascular disease (23.8% [3048/12,803]). Twenty percent of adult patients (1300/6516) were considered obese based on a body-mass index > 30 kg/m^2^. Other comorbidities reported for > 10% of patients included COPD (12.4% [1592/12,803]) and diabetes (11.6% [1490/12,803]). In addition, 21.8% (1546/7082) of adult patients were current smokers. As assessed by the Barthel Index, 83.4% (2617/3140) of patients aged ≥65 years had mild or minimal functional impairment. Over half of the included patients (56.2%) had consultations with a general practitioner within the previous 3 months, and 29.2% had been hospitalized in the previous 12 months. The overall influenza vaccination rate was 14.3%, and antivirals were used by 19.9%.

**Table 2:** Characteristics of included patients (*N* = 12,803)

**Characteristic**	**Category**	**n (%)**
Age range (y)	< 1	2063 (16.1)
	1 to < 5	2586 (20.2)
	5 to < 15	900 (7.0)
	15 to < 50	2452 (19.2)
	50 to < 65	1425 (11.1)
	65 to < 75	1241 (9.7)
	75 to < 85	1255 (9.8)
	≥85	881 (6.9)
Sex	Female	6269 (49.0)
	Male	6534 (51.0)
Chronic conditions	0	7506 (58.6)
	1	2499 (19.5)
	> 1	2798 (21.9)
Pregnant ^a^	Yes	263 (2.1)
Hospitalized in the last 12 months ^b^	Yes	3417 (29.2)
Underlying chronic conditions	Cardiovascular disease	3048 (23.8)
	COPD	1592 (12.4)
	Diabetes	1490 (11.6)
	Renal disease	745 (5.8)
	Neoplasms	669 (5.2)
	Asthma	655 (5.1)
	Neuromuscular disease	487 (3.8)
	Immunological disorders	392 (3.1)
	Autoimmune diseases	230 (1.8)
	Cirrhosis	206 (1.6)
	Rheumatological disease	135 (1.1)
Obesity ^c^	Yes	1300 (20.0)
Consultations with a general practitioner in the last 3 months	0	4848 (43.8)
	1	2187 (19.7)
	> 1	4043 (36.5)
Smoking habits ^d^	Never smoker	3583 (50.6)
	Past smoker	1953 (27.6)
	Current smoker	1546 (21.8)
Functional status impairment (Barthel score) ^e^	Total (0–15)	172 (5.5)
	Severe (20–35)	129 (4.1)
	Moderate (40–55)	222 (7.1)
	Mild (60–90)	831 (26.5)
	Minimal (95–100)	1786 (56.9)
Influenza vaccination ≥14 days from symptom onset	Yes	1818 (14.2)
Antiviral use during the current episode	Yes	2544 (19.9)

Abbreviation: *COPD* Chronic obstructive pulmonary disease

^a^ Proportion of females (*N* = 6269)

^b^*N* = 11,690

^c^ Assessed for patients aged ≥18 years only, defined as a body mass index > 30 kg/m^2^, *N* = 6516

^d^ Assessed for patients aged ≥18 years only, *N* = 7082

^e^ Assessed for patients aged ≥65 years only, *N* = 3140

Influenza vaccination rates increased with age from as low as 1.1% in patients aged < 1 year to as high as 49.0% in patients aged ≥85 years, whereas no clear trend was observed for antiviral use (Additional file 4). As expected, chronic conditions increased with age (from 2.9% in patients < 1 year to 89.2% in patients ≥80 years).

Influenza vaccination rates varied from 0.0% (Kenya) to 42.9% (France), and rates of antiviral use varied from 0.0% (Kenya and Serbia) to 93.7% (China) (Additional files 5 and 6). Demographics varied considerably between sites due to differences in the populations treated at the participating hospitals. For example, the site in China included only children, whereas the sites in Canada, Czech Republic, and France included only adults.

### Influenza strain circulation in hospitalized patients

Of the 12,803 included patients, 4306 (33.6%) tested positive for influenza virus infection by RT-PCR (Fig. 1).

**Fig. 2:**
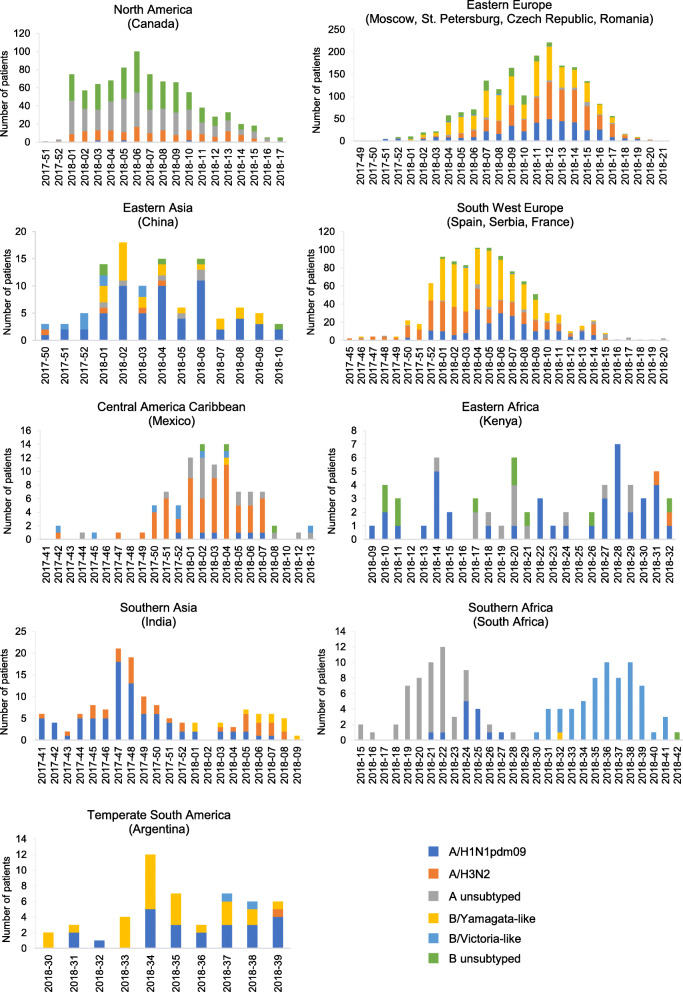
Influenza strain circulation by year-week and World Health Organization influenza transmission zone. Influenza strains were detected by RT-PCR. An individual patient could have been positive for more than one strain of influenza

**Fig. 3:**
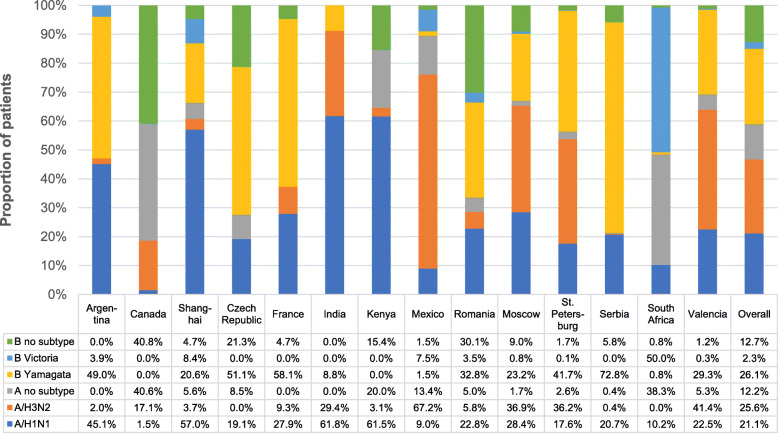
Influenza strain circulation by site and overall during 2017–2018. Influenza strains were detected by RT-PCR. An individual patient could have been positive for more than one strain of influenza

In the influenza transmission zones of North America (Canada), Eastern Europe (Moscow, St. Petersburg, Czech Republic, and Romania), East Asia (China), and South West Europe (Spain, Serbia, and France), influenza was first detected during the last few weeks of 2017 or first few weeks of 2018, after which circulation reached a single peak and then tapered off by week 10 to 15 of 2018 (Fig. 2). Influenza B/Yamagata-like, A/H1N1pdm09, and A/H3N2 viruses dominated and co-circulated in Eastern and South West Europe. In Eastern Asia, all strains co-circulated, but influenza A/H1N1pdm09 virus was dominant. Even within these transmission zones, however, strain circulation varied substantially (Fig. 3). For example, influenza B/Yamagata-like virus was common at all sites within the East Europe transmission zone, but influenza A/H3N2 virus was common only at the St. Petersburg and Moscow sites (Fig. 3 and Additional file 7). Likewise, within the South West Europe transmission zone, influenza B/Yamagata-like virus was common at all sites, but influenza A/H3N2 virus was common only in Spain (Fig. 3 and Additional file 8). Influenza A and B strains also co-circulated in North America (Canada), but subtyping was mostly unavailable at the time of the current analysis.

In the Central American Caribbean transmission zone (Mexico), the timing of seasonal influenza was similar to that of the Northern Hemisphere transmission zones. However, strain circulation differed, with influenza A/H3N2 (67.2%) virus dominating and little influenza B detected. In the Temperate South America transmission zone (Argentina), influenza circulated earlier (weeks 30 to 39 of 2017), with influenza B/Yamagata-like (49.0%) and A/H1N1pdm09 (45.1%) viruses dominating.

In contrast to the influenza transmission zones in the Northern Hemisphere, two distinct peaks of influenza activity were detected in Southern Africa (South Africa) and Southern Asia (India). In Southern Africa, a first peak, dominated by influenza A/H1N1pdm09 virus, occurred between weeks 18 and 28 of 2017, and a second, dominated by influenza A/H3N2 virus, between weeks 30 and 42 of 2017. In Southern Asia, a first peak, dominated by influenza A/H1N1pdm09 virus, occurred between week 41 of 2017 and week 1 of 2018 and a second, dominated by influenza A/H3N2 virus, between weeks 3 and 9 of 2018. No discernable peak of influenza circulation was detected in Eastern Africa (Kenya), although influenza A/H1N1pdm09 virus dominated.

Overall, across all regions of the GIHSN, influenza B/Yamagata-like virus (26.1% of influenza-positive patients) and influenza A/H3N2 (25.6%) were the most frequently detected strains, closely followed by A/H1N1pdm09 (21.1%). Influenza B/Victoria-like virus was detected in 2.3% of influenza-positive patients, unsubtyped influenza A virus in 12.2%, and influenza B of unknown lineage in 12.7%. Except in Mexico and South Africa, influenza B/Yamagata-like was the dominant B lineage detected in all countries where lineage was determined.

### Complicated hospitalization in influenza-positive patients

**Fig. 4:**
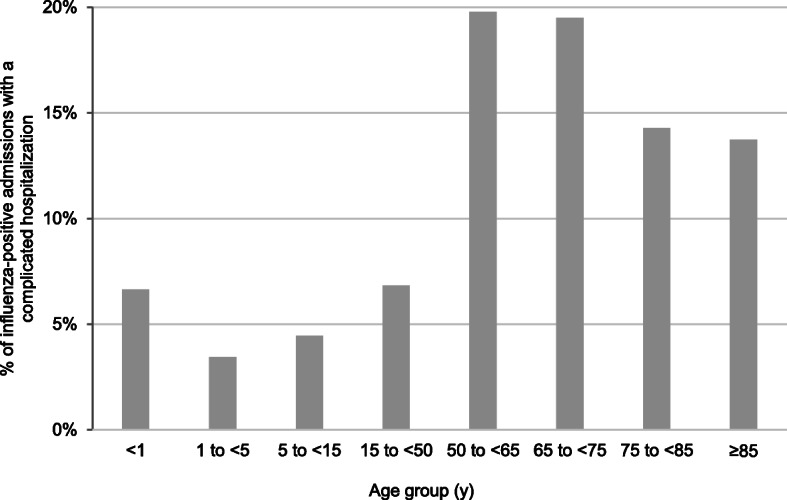
Proportion of influenza-positive patients with complicated hospitalization by age group. Complicated hospitalization was defined as ICU admission, mechanical ventilation, or death

Of the 4306 influenza-positive patients, 458 (10.6%) had a complicated hospitalization, as defined by admission to an ICU, need for mechanical ventilation, or death during hospitalization (Fig. 4). Most complicated hospitalizations were in patients aged ≥50 years. Age distributions of ICU admission (*n* = 355) and mechanical ventilation (*n* = 221) were similar, whereas death (*n* = 166) continuously increased with age (Additional file 9).

**Fig. 5:**
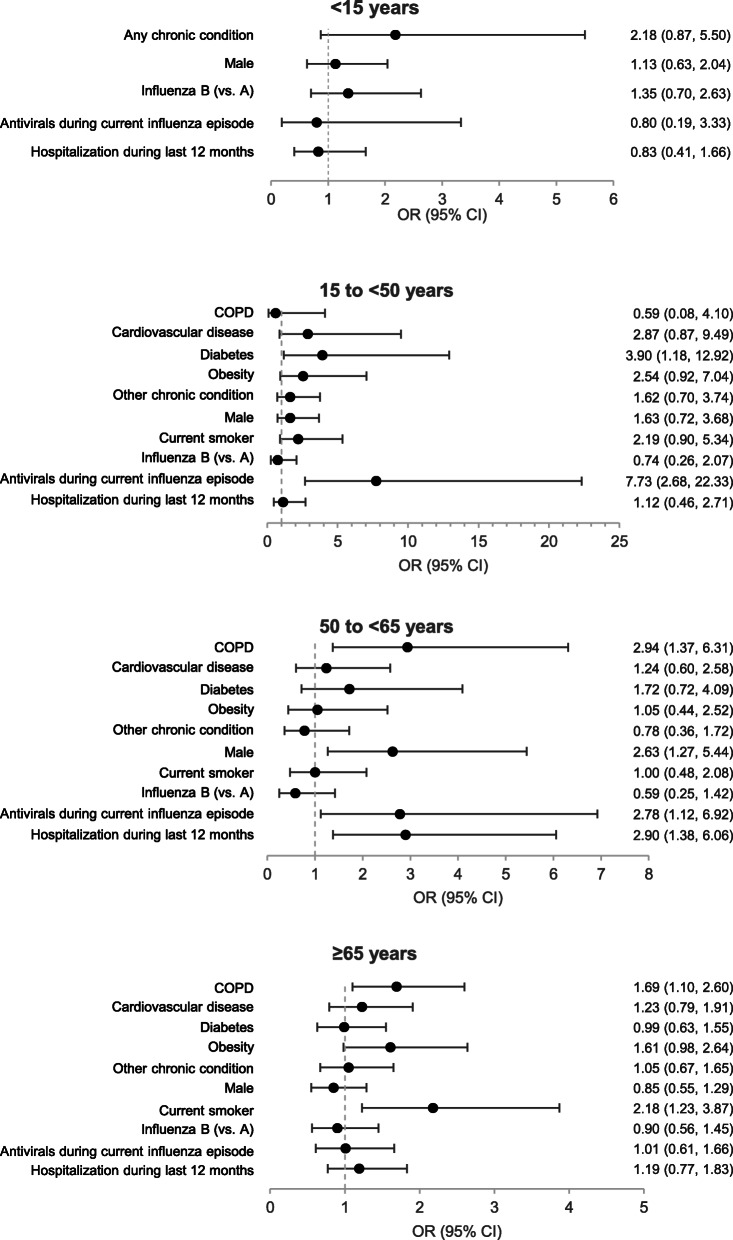
Adjusted odds ratios for complicated hospitalization in influenza-positive patients. Adjusted odds ratios for complicated hospitalization were determined in influenza-positive patients aged < 15 years (*N* = 1309), 15 to < 65 years (*N* = 1193), and ≥ 65 years (*N* = 776) by mixed effects logistic regression adjusted for age and vaccination for influenza during the previous 12 months and with site as a random effect. Complicated hospitalization was defined as ICU admission, mechanical ventilation, or death. Abbreviations: COPD, chronic obstructive pulmonary disease; CVD, cardiovascular disease

COPD was associated with complicated hospitalization in influenza-positive admissions aged 50 to < 65 years (OR, 2.94 [95% CI, 1.37–6.31]) and ≥ 65 years (1.69 [95% CI, 1.10–2.60]) (Fig. 5). Prescription of antivirals during the current influenza episode was associated with complicated hospitalization in influenza-positive admissions aged 15 to < 50 years (OR, 7.73 [95% CI, 2.68–22.33]) and 50 to < 65 years (OR, 2.78 [95% CI, 1.12–6.92]). Other factors associated with complicated hospitalization included diabetes in influenza-positive admissions aged 15 to < 50 years (OR, 3.90 [95% CI, 1.18–12.92]); male sex (OR, 2.63 [95% CI, 1.27–5.44]) and hospitalization during the last 12 months (OR, 2.90 [95% CI, 1.38–6.06]) in influenza-positive admissions aged 50 to < 65 years; and current smoking in influenza-positive admissions aged ≥65 years (OR, 2.18 [95% CI, 1.23–3.87]). No factors were found to be associated with complicated hospitalization among influenza-positive patients aged < 15 years. The frequency of complicated hospitalization did not differ between influenza A and B for any age group. The frequency of complicated hospitalization also did not differ between A/H1N1pdm09 and A/H3N2 for any age group (data not shown). All factors examined in relation to complicated hospitalization are provided in Additional file 10.

### Length of hospital stay

**Fig. 6:**
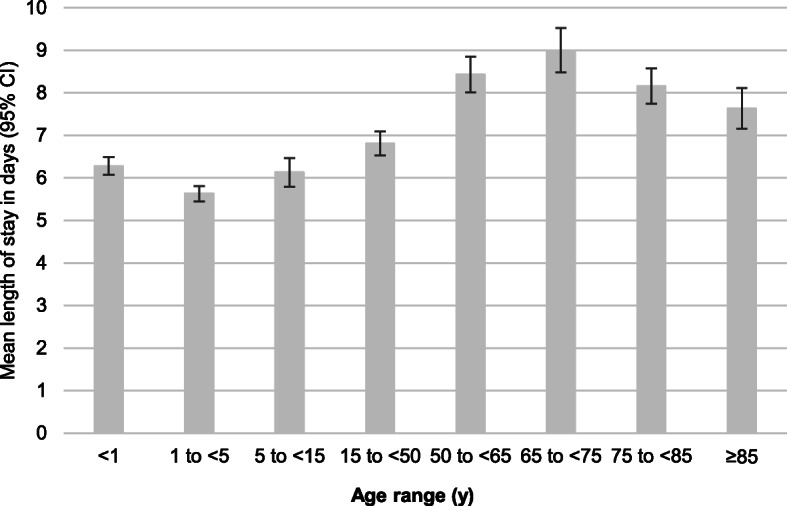
Mean length of hospital stay in influenza-positive patients. Abbreviation: CI, confidence interval

**Fig. 7:**
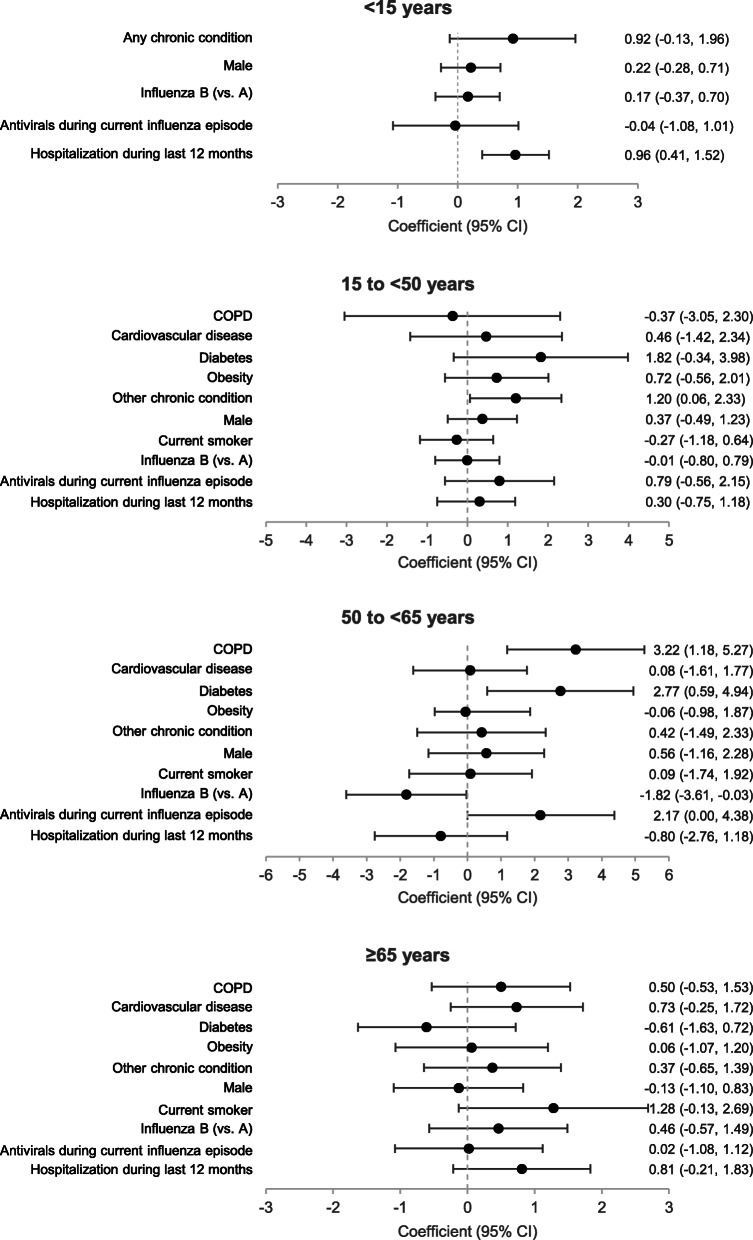
Adjusted coefficients for long hospital stays in influenza-positive patients. Coefficient estimates for length of hospital stay were determined in influenza-positive patients aged < 15 years (*N* = 1306), 15 to < 65 years (*N* = 1189), and ≥ 65 years (*N* = 775) by linear mixed-effects regression model adjusted for age and vaccination status and with site as a random effect. The coefficient indicates the change in length of hospital stay in days when the indicated factor is changed by one unit (i.e. from yes to no). Abbreviations: COPD, chronic obstructive pulmonary disease; CVD, cardiovascular disease

The mean length of hospital stay in influenza-positive admissions was lowest in admissions aged < 15 years (approximately 6 days) and highest in admissions aged 65–74 years (approximately 9 days) (Fig. 6). COPD (coefficient, 3.22 [95% CI, 1.18–5.27]) and diabetes (coefficient, 2.77 [95% CI, 0.59–4.94]) were associated with a longer hospital stay and influenza B (vs. A) with a shorter hospital stay (coefficient, − 1.82 [− 3.61−− 0.03]) in influenza-positive admissions aged 50 to < 65 years, and other chronic conditions (coefficient, 1.20 [95% CI, 0.06–2.33]) were associated with a longer hospital stay in influenza-positive admissions aged 15 to < 50 years (Fig. 7). Hospitalization during the previous 12 months was associated with a longer hospital stay in influenza-positive admissions aged < 15 years (coefficient, 0.96 [95% CI, 0.41–1.52]). No factors associated with a longer hospital stay were identified in influenza-positive admissions aged ≥65 years. Length of hospital stay did not differ between influenza A and B for any age group. The length of hospital stay also did not differ between A/H1N1pdm09 and A/H3N2 for any age group (data not shown).

## Discussion

The current study showed that during the 2017–2018 influenza season, approximately 10% of all hospitalized cases of influenza virus infection were complicated, as defined by admission to an ICU, need for mechanical ventilation, or death. Factors associated with complicated hospitalization in patients with influenza virus infection included COPD, diabetes, and hospitalization during the previous 12 months, although male sex, cardiovascular disease, and some lifestyle factors (smoking and obesity) were also identified in certain subgroups. Age was also an important factor associated with complicated hospitalization and a longer hospital stay in patients with influenza virus infection. Most of these are known risk factors for severe influenza illness [4]. In addition to these factors, prescription of antiviral medication during the current episode was associated with an increased frequency of complications, probably because antivirals were mostly prescribed for influenza cases with a high likelihood of developing complications.

Influenza A infections are often thought to result in more severe illness than influenza B infections [15]. The current study, however, did not find a difference between influenza A and B or between A/H1N1pdm09 and A/H3N2 in the risk for complicated hospitalization. It also did not find a consistent difference between influenza A and B in the risk for individual components of complicated hospitalization or in the length of hospital stay: influenza B was associated with shorter hospital stay in influenza-positive admissions 50 to < 65 years of age but a higher risk for mechanical ventilation in those under 15 years of age. These findings support a systematic literature review concluding that clinical presentation and severity of influenza illness do not appear to differ between influenza strains [16].

During the 2017–2018 influenza season, influenza accounted for approximately one-third of hospital admissions with influenza-like symptoms, which agrees with previous influenza seasons in the GISHN, where proportions were between 21 and 31% [8–10, 17]. As in previous studies, all strains of influenza were detected, with widely varying strain circulation between sites and even neighboring regions. Influenza B/Yamagata was the most frequently detected influenza strain, although A/H3N2 and A/H1N1pdm09 were nearly as common. There were some minor differences in timing, although these three strains essentially co-circulated globally. Furthermore, although a B/Victoria-lineage strain was included in the 2017–2018 Northern Hemisphere and 2018 Southern Hemisphere trivalent influenza vaccines, Yamagata was the dominant lineage of influenza B [18, 19]. Finally, as in previous studies, more than half of the patients hospitalized with influenza virus infection did not have known chronic conditions, highlighting that influenza can cause severe illness even in individuals without high-risk conditions.

By using a shared protocol combined with active, prospective surveillance across many countries and continents, the GIHSN provides a combination of extensive global virological data and clinical data on severe seasonal influenza illness. Data from the GIHSN can be used to identify associations between influenza virology/epidemiology, patient characteristics, and severe cases of influenza illness. In the current study, we pooled data from more than 4000 influenza-positive patients at 14 coordinating sites in 13 countries on four continents. Although this provided a large enough dataset to assess many factors potentially associated with complications, the influence of some factors known to be associated with severe influenza, such as human immunodeficiency virus infection and neurological conditions [4], could not be assessed because related information was not collected.

Analyzing pooled surveillance data can be complicated by differences in clinical practice, case definitions, and procedures between sites, as well as patients’ health-seeking behaviors and access to care and vaccination. The GIHSN improves comparability between sites by using active surveillance, validated RT-PCR, and a common core protocol for identifying hospitalized cases of influenza illness. In addition, to reduce bias caused by false-negative tests due to decreasing viral shedding over time [8], only patients who had been hospitalized within 7 days of symptom onset were eligible. To further reduce the effects of bias from differences between sites, factors associated with complicated influenza-related hospitalization were identified using a mixed effects model with a random effect per site [20, 21]. These steps represent improvements over many surveillance efforts, where interpretability may be limited by non-systematic sampling, incomplete case ascertainment, lack of adjustment for confounders, sparse numbers, lack of consensus about case definitions and risk factors, and a lack of comparison groups. Despite the efforts to reduce heterogeneity, CIs were wide, which limited the ability to detect certain factors and interpret the size of the effects.

We did not examine the effect of influenza vaccination on complications or length of hospital stay because of low vaccination rates; the overall influenza vaccination rate across all sites and all included patients was below 15%, and, for more than half of the sites, the rate of influenza vaccination was below 10%, although a few sites had rates above 30%. This highlights differences in clinical practice, access to care, and included populations across the different sites.

Our results should be interpreted in the context of the definition for complicated hospitalization, as well as the case definition for influenza. There is currently no consensus of how to define complicated hospitalization, although some studies have reported on ICU admission, mechanical ventilation, and in-hospital death [4, 16, 22, 23]. In the current study, complicated hospitalization was a composite of three outcomes, one of which is a treatment or support modality (mechanical ventilation), one mostly a metric for health care utilization (ICU admission), and one an indicator of severity (death during hospitalization). These, as well as length of hospitalization, capture different underlying aspects of severity, and each is influenced by a variety of patient, cultural, and healthcare practice factors, which may complicate interpreting results for individual factors. Nonetheless, this study was able to identify some common factors associated with complicated hospitalization and a longer hospital stay in influenza-positive patients. Given the limitations of influenza-like-illness case definitions for predicting influenza in individuals, particularly in older adults, to identify the maximum number of hospitalized influenza cases, potential cases were identified based on the presence of any acute respiratory symptoms possibly associated with influenza rather than based on a specific influenza-like illness case definition. Although this would reduce specificity of the inclusion criteria, specificity was ensured by confirming influenza virus infection by RT-PCR.

Care should be taken when generalizing the findings of this study. Although substantial efforts were made to improve comparability of data, the current results represent a single season and depend on the strains, sites, and populations included.

## Conclusions

This study showed that more than one in ten hospitalized cases of influenza virus infections detected during the 2017–2018 influenza season was complicated by need for mechanical ventilation, admission to an ICU, or death. Common factors associated with an increased risk of severe influenza were also found to be associated with complicated or longer hospitalization in these patients, but the study did not find differences between influenza strains. The study also provides further evidence that influenza can cause severe illness even in individuals without high-risk conditions. Further studies are needed to determine how other factors, such as health-seeking behaviors, access to care, access to vaccination, and appropriate use of antivirals influence complicated hospitalization due to influenza virus infection.

## Supplementary information

**Additional file 1: Supplemental Table 1.** Admission diagnoses possibly associated with an influenza infection in patients ≥5 years of age.

**Additional file 2: Supplemental Table 2.** Admission diagnoses possibly associated with an influenza infection in patients < 5 years of age.

**Additional file 3: Supplemental Table 3.** Sample collection.

**Additional file 4: Supplemental Table 4.** Characteristics of included patients by age group

**Additional file 5: Supplemental Table 5.** Characteristics of influenza-positive patients by site (Argentina, Canada, China, Czech Republic, France, India, and Kenya).

**Additional file 6: Supplemental Table 6.** Characteristics of influenza-positive patients by site (Mexico, Romania, Moscow, St. Petersburg, Serbia, South Africa, and Spain).

**Additional file 7: Supplemental Figure 1.** Influenza strain circulation by year-week for individual sites within the East Europe influenza transmission zone.

**Additional file 8: Supplemental Figure 2.** Description of data: Influenza strain circulation by year-week for individual sites within the South West Europe influenza transmission zone.

**Additional file 9: Supplemental Figure 3.** Proportion of influenza-positive patients admitted to an ICU, requiring mechanical ventilation, or that died while hospitalized by age group.

**Additional file 10: Supplemental Table 7.** Factors associated with ICU admission, mechanical ventilation, and death during hospitalization in influenza-positive patients.

## Data Availability

The data supporting the conclusions of this article are included within the article and its additional files. Patient-level data are not publicly available, but outputs from additional analyses can be requested by writing foundationforinfluenza@gihsn.org.

## Abbreviations

CI: Confidence interval
COPD: Chronic obstructive pulmonary disease
GIHSN: Global influenza hospital surveillance network
ICU: Intensive care unit
OR: Odds ratio
RT-PCR: Reverse transcription-polymerase chain reaction

## References

[1] World Health Organization: Influenza (Seasonal). https://www.who.int/en/news-room/fact-sheets/detail/influenza-(seasonal) . Accessed 02 Jul 2019. (2012).

[2] Iuliano AD, Roguski KM, Chang HH, Muscatello DJ, Palekar R, Tempia S (2018). Estimates of global seasonal influenza-associated respiratory mortality: a modelling study. Lancet.

[3] World Health Organization (2012). Vaccines against influenza WHO position paper - November 2012. Wkly Epidemiol Rec.

[4] Coleman BL, Fadel SA, Fitzpatrick T, Thomas SM (2018). Risk factors for serious outcomes associated with influenza illness in high- versus low- and middle-income countries: systematic literature review and meta-analysis. Influenza Other Respir Viruses.

[5] Puig-Barbera J, Tormos A, Sominina A, Burtseva E, Launay O, Ciblak MA (2014). First-year results of the global influenza hospital surveillance network: 2012-2013 northern hemisphere influenza season. BMC Public Health.

[6] Blanton L, Dugan VG, Abd Elal AI, Alabi N, Barnes J, Brammer L (2019). Update: influenza activity - United States, September 30, 2018-February 2, 2019. MMWR Morb Mortal Wkly Rep.

[7] GIHSN: Global Influenza Hospital Surveillance Network. https://www.gihsn.org/ . Accessed 10 Apr 2019. (2019).

[8] Puig-Barbera J, Natividad-Sancho A, Trushakova S, Sominina A, Pisareva M, Ciblak MA (2016). Epidemiology of hospital admissions with influenza during the 2013/2014 northern hemisphere influenza season: results from the global influenza hospital surveillance network. PLoS One.

[9] Puig-Barbera J, Burtseva E, Yu H, Cowling BJ, Badur S, Kyncl J (2016). Influenza epidemiology and influenza vaccine effectiveness during the 2014–2015 season: annual report from the global influenza hospital surveillance network. BMC Public Health.

[10] Baselga-Moreno V, Trushakova S, McNeil S, Sominina A, Nunes MC, Draganescu A (2019). Influenza epidemiology and influenza vaccine effectiveness during the 2016-2017 season in the global influenza hospital surveillance network (GIHSN). BMC Public Health.

[11] GIHSN: Global Influenza Hospital-based Surveillance Network (GIHSN) Core Protocol. https://www.gihsn.org/images/gihsn/GIHSN-Core-Protocol-June-2014.pdf . Accessed 10 Apr 2019 (2013).

[12] Mahoney FI, Barthel DW (1965). Functional evaluation: the Barthel index. Md State Med J.

[13] World Health Organization: Influenza. https://www.who.int/immunization/monitoring_surveillance/burden/vpd/WHO_SurveillanceVaccinePreventable_09_Influenza_R2.pdf?ua=1 . Accessed 10 Dec 2019. (2018).

[14] World Health Organization: Influenza transmission zones. https://www.who.int/csr/disease/swineflu/Influenza_transmission_zones.pdf?ua=1 . Accessed 02 Sep 2019 (2011).

[15] Su S, Chaves SS, Perez A, D'Mello T, Kirley PD, Yousey-Hindes K (2014). Comparing clinical characteristics between hospitalized adults with laboratory-confirmed influenza a and B virus infection. Clin Infect Dis.

[16] Caini S, Kroneman M, Wiegers T, El Guerche-Seblain C, Paget J (2018). Clinical characteristics and severity of influenza infections by virus type, subtype, and lineage: a systematic literature review. Influenza Other Respir Viruses.

[17] Puig-Barbera J, Natividad-Sancho A, Launay O, Burtseva E, Ciblak MA, Tormos A (2014). 2012-2013 seasonal influenza vaccine effectiveness against influenza hospitalizations: results from the global influenza hospital surveillance network. PLoS One.

[18] World Health Organization: Recommended composition of influenza virus vaccines for use in the 2017–2018 northern hemisphere influenza season. https://www.who.int/influenza/vaccines/virus/recommendations/2017_18_north/en/ . Accessed 03 Sep 2019 (2017).

[19] World Health Organization: Recommended composition of influenza virus vaccines for use in the 2018 southern hemisphere influenza season. https://www.who.int/influenza/vaccines/virus/recommendations/2018_south/en/. Accessed 03 Sep 2019 (2017).

[20] Jaeger TF (2008). Categorical data analysis: away from ANOVAs (transformation or not) and towards Logit mixed models. J Mem Lang.

[21] Beitler PJ, Landis JR (1985). A mixed-effects model for categorical data. Biometrics.

[22] Martinez A, Soldevila N, Romero-Tamarit A, Torner N, Godoy P, Rius C (2019). Risk factors associated with severe outcomes in adult hospitalized patients according to influenza type and subtype. PLoS One.

[23] Nichols MK, Andrew MK, Hatchette TF, Ambrose A, Boivin G, Bowie W (2018). Influenza vaccine effectiveness to prevent influenza-related hospitalizations and serious outcomes in Canadian adults over the 2011/12 through 2013/14 influenza seasons: a pooled analysis from the Canadian immunization research network (CIRN) serious outcomes surveillance (SOS network). Vaccine..

