# Supplementary material for: Complicated hospitalization due to influenza: results from the Global Hospital Influenza Network for the 2017–2018 season
Source: BMC Infect Dis. 2020 Jul 2;20:465. doi: 10.1186/s12879-020-05167-4 (PMC7330273; doi:10.1186/s12879-020-05167-4)
Supplement: Supplementary file 2 — Additional file 2: Supplemental Table 2. Admission diagnoses possibly associated with an influenza infection in patients < 5 years of age. [file 12879_2020_5167_MOESM2_ESM.docx]

**Supplemental Table 2. Admission diagnoses possibly associated with an influenza infection in patients < 5 years of age**

| **Diagnosis** | **ICD 9 Codes** | **ICD 10 Codes** |
| --- | --- | --- |
| Acute upper or lower respiratory disease | 382.9; 460 to 466 | J00-J06, J20-J22 |
| Dyspnea, breathing anomaly, shortness of breath, tachypnea | 786.0; 786.00; 786.05-786.07; 786.09; 786.9 | R06.0, R06, R06.9, R06.3, R06.00, R06.09, R06.83, R06.02, R06.82, R06.2, R06.89 |
| Asthma | 493-493.92 | J45.2-J45.22, J45.9-J45.998, J44-J44.9 |
| Pneumonia and influenza | 480 to 488 | J09-J18 |
| Heart failure | 428-429.0 | I50-I50.9; I51.4 |
| Myalgia | 729.1 | M79.1 |
| Altered consciousness, convulsions, febrile convulsions | 780.01-780.02; 780.09; 780.31- 780.32 | R40.20, R40.4, R40.0, R40.1, R56.00, R56.01 |
| Fever or fever unknown origin or non-specified | 780.6-780.60 | R50, R50.9 |
| Cough | 786.2 | R05 |
| Gastrointestinal manifestations | 009.0; 009.3 | A09.0; A09.9 |
| Sepsis, systemic inflammatory response syndrome | 995.90-995.94 | R65.10, R65.11, R65.20, A41.9 |
