# Supplementary material for: Complicated hospitalization due to influenza: results from the Global Hospital Influenza Network for the 2017–2018 season
Source: BMC Infect Dis. 2020 Jul 2;20:465. doi: 10.1186/s12879-020-05167-4 (PMC7330273; doi:10.1186/s12879-020-05167-4)
Supplement: Supplementary file 3 — Additional file 3: Supplemental Table 3. Sample collection. [file 12879_2020_5167_MOESM3_ESM.docx]

**Supplemental Table 3. Sample collection**

| **Site** | **Patients aged <14 years** | **Patients aged ≥14 years** |
| --- | --- | --- |
| Argentina | Nasopharyngeal swab + nasal swab | Nasopharyngeal swab + oral or pharyngeal swab |
| Canada | N/A | Nasopharyngeal swab + BAL or endotracheal aspirate for patients admitted to an ICU |
| China | Nasopharyngeal aspirate | N/A |
| Czech Republic | N/A | Nasopharyngeal swab + oral or pharyngeal swab |
| France | N/A | Nasopharyngeal swab |
| India | Nasopharyngeal swab + nasal swab | Nasopharyngeal swab + oral or pharyngeal swab |
| Kenya | Nasopharyngeal swab + oropharyngeal swab | Nasopharyngeal swab + oropharyngeal swab |
| Mexico | Two nasopharyngeal swabs (one from each nostril) | Two nasopharyngeal swabs |
| Romania | Nasopharyngeal swab + nasal swab | Nasopharyngeal swab + oral or pharyngeal swab |
| Moscow | Nasopharyngeal swab + nasal swab | Nasopharyngeal swab + oral or pharyngeal swab |
| St Petersburg | Nasopharyngeal swab + nasal swab | Nasopharyngeal swab + oral or pharyngeal swab |
| Serbia | Nasopharyngeal swab + nasal swab | Nasopharyngeal swab + oral or pharyngeal swab |
| Spain | Nasopharyngeal swab + nasal swab | Nasopharyngeal swab + pharyngeal swab |
| South Africa | Nasopharyngeal swab | Nasal swab + oral swab |

Abbreviation: N/A, not applicable because the site did not include the population
