# Supplementary material for: Complicated hospitalization due to influenza: results from the Global Hospital Influenza Network for the 2017–2018 season
Source: BMC Infect Dis. 2020 Jul 2;20:465. doi: 10.1186/s12879-020-05167-4 (PMC7330273; doi:10.1186/s12879-020-05167-4)
Supplement: Supplementary file 4 — Additional file 4: Supplemental Table 4. Characteristics of included patients by age group [file 12879_2020_5167_MOESM4_ESM.docx]

**Supplemental Table 4. Characteristics of included patients by age group**

| **Characteristic** | **Category** | **n (%)** | | | | | | | |
| --- | --- | --- | --- | --- | --- | --- | --- | --- | --- |
|  |  | **<1 y** | **1 to <5 y** | **5 to <15 y** | **15 to <50 y** | **50 to <65 y** | **65 to <75 y** | **75 to <85 y** | **≥85 y** |
| Sex |  | N=2063 | N=2586 | N=900 | N=2452 | N=1425 | N=1241 | N=1255 | N=881 |
|  | Female | 847 (41.1) | 1112 (43.0) | 397 (44.1) | 1486 (60.6) | 750 (52.6) | 572 (46.1) | 587 (46.8) | 518 (58.8) |
|  | Male | 1216 (58.9) | 1474 (57.0) | 503 (55.9) | 966 (39.4) | 675 (47.4) | 669 (53.9) | 668 (53.2) | 363 (41.2) |
| Chronic conditions |  | N=2063 | N=2586 | N=900 | N=2452 | N=1425 | N=1241 | N=1255 | N=881 |
|  | 0 | 2003 (97.1) | 2400 (92.8) | 768 (85.3) | 1635 (66.7) | 360 (25.3) | 151 (12.2) | 99 (7.9) | 90 (10.2) |
|  | 1 | 54 (2.6) | 162 (6.3) | 118 (13.1) | 585 (23.9) | 564 (39.6) | 394 (31.7) | 347 (27.6) | 275 (31.2) |
|  | > 1 | 6 (0.3) | 24 (0.9) | 14 (1.6) | 232 (9.5) | 501 (35.2) | 696 (56.1) | 809 (64.5) | 516 (58.6) |
| Hospitalized within the last 12 months |  | N=2044 | N=2569 | N=876 | N=2306 | N=1242 | N=1003 | N=981 | N=669 |
|  | Yes | 589 (28.8) | 903 (35.1) | 225 (25.7) | 406 (17.6) | 371 (29.9) | 332 (33.1) | 360 (36.7) | 231 (34.5) |
| Underlying chronic conditions |  | N=2063 | N=2586 | N=900 | N=2452 | N=1425 | N=1241 | N=1255 | N=881 |
|  | Cardiovascular disease | 24 (1.2) | 41 (1.6) | 15 (1.7) | 189 (7.7) | 529 (37.1) | 727 (58.6) | 868 (69.2) | 655 (74.3) |
|  | COPD | 18 (0.9) | 35 (1.4) | 9 (1.0) | 101 (4.1) | 353 (24.8) | 416 (33.5) | 438 (34.9) | 222 (25.2) |
|  | Asthma | 4 (0.2) | 53 (2.0) | 49 (5.4) | 165 (6.7) | 117 (8.2) | 94 (7.6) | 114 (9.1) | 59 (6.7) |
|  | Diabetes | 0 (0.0) | 2 (0.1) | 6 (0.7) | 107 (4.4) | 291 (20.4) | 396 (31.9) | 436 (34.7) | 252 (28.6) |
|  | Immunological disorders | 4 (0.2) | 23 (0.9) | 15 (1.7) | 156 (6.4) | 79 (5.5) | 61 (4.9) | 45 (3.6) | 9 (1.0) |
|  | Rheumatological disorders | 1 (<0.1) | 0 (0.0) | 2 (0.2) | 31 (1.3) | 30 (2.1) | 35 (2.8) | 23 (1.8) | 13 (1.5) |
|  | Renal disease | 4 (0.2) | 6 (0.2) | 8 (0.9) | 109 (4.4) | 100 (7.0) | 136 (11.0) | 208 (16.6) | 174 (19.8) |
|  | Neuromuscular disorders | 10 (0.5) | 45 (1.7) | 34 (3.8) | 58 (2.4) | 53 (3.7) | 76 (6.1) | 111 (8.8) | 100 (11.4) |
|  | Cirrhosis | 0 (0.0) | 1 (<0.1) | 2 (0.2) | 56 (2.3) | 59 (4.1) | 45 (3.6) | 33 (2.6) | 10 (1.1) |
|  | Neoplasm | 0 (0.0) | 5 (0.2) | 2 (0.2) | 81 (3.3) | 131 (9.2) | 178 (14.3) | 169 (13.5) | 103 (11.7) |
|  | Autoimmune disorders | 2 (0.1) | 1 (<0.1) | 6 (0.7) | 75 (3.1) | 52 (3.6) | 45 (3.6) | 31 (2.5) | 18 (2.0) |
| Obesity ^a^ |  |  |  |  | N=2221 | N=1234 | N=1123 | N=1139 | N=799 |
|  | Yes | - | - | - | 252 (11.3) | 320 (25.9) | 286 (25.5) | 268 (23.5) | 174 (21.8) |
| Outpatient consultations last 3 months |  | N=1809 | N=2396 | N=846 | N=2269 | N=1190 | N=945 | N=959 | N=664 |
|  | 0 | 866 (47.9) | 815 (34.0) | 392 (46.3) | 1348 (59.4) | 548 (46.1) | 302 (32.0) | 320 (33.4) | 257 (38.7) |
|  | 1 | 324 (17.9) | 778 (32.5) | 249 (29.4) | 305 (13.4) | 178 (15.0) | 153 (16.2) | 119 (12.4) | 81 (12.2) |
|  | >1 | 619 (34.2) | 803 (33.5) | 205 (24.2) | 616 (27.1) | 464 (39.0) | 490 (51.9) | 520 (54.2) | 326 (49.1) |
| Smoking habits ^a^ |  |  |  |  | N=2417 | N=1394 | N=1208 | N=1221 | N=842 |
|  | Never smoker | - | - | - | 1422 (58.8) | 554 (39.7) | 469 (38.8) | 583 (47.7) | 555 (65.9) |
|  | Past smoker | - | - | - | 340 (14.1) | 381 (27.3) | 470 (38.9) | 507 (41.5) | 255 (30.3) |
|  | Current smoker | - | - | - | 655 (27.1) | 459 (32.9) | 269 (22.3) | 131 (10.7) | 32 (3.8) |
| Functional status impairment (Barthel Index) ^b^ |  |  |  |  |  |  | N=1144 | N=1163 | N=833 |
|  | Total (0-15) | - | - | - | - | - | 33 (2.9) | 47 (4.0) | 92 (11.0) |
|  | Severe (20-35) | - | - | - | - | - | 37 (3.2) | 45 (3.9) | 47 (5.6) |
|  | Moderate (40-55) | - | - | - | - | - | 67 (5.9) | 71 (6.1) | 84 (10.1) |
|  | Mild (60-90) | - | - | - | - | - | 224 (19.6) | 308 (26.5) | 299 (35.9) |
|  | Minimal (95-100) | - | - | - | - | - | 783 (68.4) | 692 (59.5) | 311 (37.3) |
| Influenza vaccination ≥14 days from symptom onset |  | N=2063 | N=2586 | N=900 | N=2452 | N=1425 | N=1241 | N=1255 | N=881 |
|  | Yes | 23 (1.1) | 90 (3.5) | 80 (8.9) | 153 (6.2) | 190 (13.3) | 348 (28.0) | 502 (40.0) | 432 (49.0) |
| Antiviral use during the current episode |  | N=2063 | N=2586 | N=900 | N=2452 | N=1425 | N=1241 | N=1255 | N=881 |
|  | Yes | 2544 (19.9) | 295 (14.3) | 429 (16.6) | 164 (18.2) | 393 (16.0) | 339 (23.8) | 329 (26.5) | 352 (28.0) |

Abbreviation: COPD, chronic obstructive pulmonary disease

^a^ Patients aged ≥18 years only

^b^ Patients aged ≥65 years only
