# Supplementary material for: Complicated hospitalization due to influenza: results from the Global Hospital Influenza Network for the 2017–2018 season
Source: BMC Infect Dis. 2020 Jul 2;20:465. doi: 10.1186/s12879-020-05167-4 (PMC7330273; doi:10.1186/s12879-020-05167-4)
Supplement: Supplementary file 5 — Additional file 5: Supplemental Table 5. Characteristics of influenza-positive patients by site (Argentina, Canada, China, Czech Republic, France, India, and Kenya). [file 12879_2020_5167_MOESM5_ESM.docx]

**Supplemental Table 5. Characteristics of influenza-positive patients by site (Argentina, Canada, China, Czech Republic, France, India, and Kenya)**

|  |  | **n (%)** | | | | | | |
| --- | --- | --- | --- | --- | --- | --- | --- | --- |
| **Characteristic** | **Category** | **Argentina** | **Canada** | **China** | **Czech Republic** | **France** | **India** | **Kenya** |
| Age |  | N=73 | N=1026 | N=399 | N=117 | N=98 | N=609 | N=386 |
|  | <1 y | 19 (26.0) | 0 (0.0) | 228 (57.1) | 0 (0.0) | 0 (0.0) | 14 (2.3) | 149 (38.6) |
|  | 1 to <5 y | 17 (23.3) | 0 (0.0) | 149 (37.3) | 0 (0.0) | 0 (0.0) | 21 (3.4) | 194 (50.3) |
|  | 5 to <15 y | 24 (32.9) | 0 (0.0) | 22 (5.5) | 0 (0.0) | 0 (0.0) | 9 (1.5) | 42 (10.9) |
|  | 15 to <50 y | 7 (9.6) | 139 (13.5) | 0 (0.0) | 39 (33.3) | 18 (18.4) | 181 (29.7) | 0 (0.0) |
|  | 50 to <65 y | 3 (4.1) | 178 (17.3) | 0 (0.0) | 24 (20.5) | 24 (24.5) | 183 (30.0) | 0 (0.0) |
|  | 65 to <75 y | 2 (2.7) | 233 (22.7) | 0 (0.0) | 31 (26.5) | 18 (18.4) | 122 (20.0) | 1 (0.3) |
|  | 75 to <85 y | 1 (1.4) | 268 (26.1) | 0 (0.0) | 17 (14.5) | 23 (23.5) | 62 (10.2) | 0 (0.0) |
|  | ≥85 y | 0 (0.0) | 208 (20.3) | 0 (0.0) | 6 (5.1) | 15 (15.3) | 17 (2.8) | 0 (0.0) |
| Sex |  | N=73 | N=1026 | N=399 | N=117 | N=98 | N=609 | N=386 |
|  | Female | 35 (47.9) | 551 (53.7) | 151 (37.8) | 57 (48.7) | 51 (52.0) | 284 (46.6) | 157 (40.7) |
|  | Male | 38 (52.1) | 475 (46.3) | 248 (62.2) | 60 (51.3) | 47 (48.0) | 325 (53.4) | 229 (59.3) |
| Chronic Conditions |  | N=73 | N=1026 | N=399 | N=117 | N=98 | N=609 | N=386 |
|  | 0 | 48 (65.8) | 108 (10.5) | 396 (99.2) | 39 (33.3) | 16 (16.3) | 149 (24.5) | 361 (93.5) |
|  | 1 | 23 (31.5) | 229 (22.3) | 3 (0.8) | 39 (33.3) | 37 (37.8) | 246 (40.4) | 25 (6.5) |
|  | >1 | 2 (2.7) | 689 (67.2) | 0 (0.0) | 39 (33.3) | 45 (45.9) | 214 (35.1) | 0 (0.0) |
| Hospitalized within the last 12 months |  | - | - | N=399 | N=117 | N=98 | N=609 | N=386 |
|  | Yes | - | - | 69 (17.3) | 32 (27.4) | 42 (42.9) | 194 (31.9) | 36 (9.3) |
| Underlying chronic conditions |  | N=73 | N=1026 | N=399 | N=117 | N=98 | N=609 | N=386 |
|  | Cardiovascular disease | 2 (2.7) | 747 (72.8) | 3 (0.8) | 49 (41.9) | 27 (27.6) | 222 (36.5) | 2 (0.5) |
|  | COPD | 5 (6.8) | 269 (26.2) | 0 (0.0) | 7 (6.0) | 30 (30.6) | 146 (24.0) | 1 (0.3) |
|  | Asthma | 4 (5.5) | 129 (12.6) | 0 (0.0) | 4 (3.4) | 15 (15.3) | 12 (2.0) | 7 (1.8) |
|  | Diabetes | 3 (4.1) | 309 (30.1) | 0 (0.0) | 24 (20.5) | 22 (22.4) | 128 (21.0) | 1 (0.3) |
|  | Immunological disorders | 13 (17.8) | 108 (10.5) | 0 (0.0) | 17 (14.5) | 5 (5.1) | 16 (2.6) | 4 (1.0) |
|  | Rheumatological disorders | 0 (0.0) | 52 (5.1) | 0 (0.0) | 3 (2.6) | 12 (12.2) | 9 (1.5) | 0 (0.0) |
|  | Renal disease | 0 (0.0) | 144 (14.0) | 0 (0.0) | 7 (6.0) | 16 (16.3) | 51 (8.4) | 1 (0.3) |
|  | Neuromuscular disorders | 0 (0.0) | 149 (14.5) | 0 (0.0) | 1 (0.9) | 3 (3.1) | 33 (5.4) | 9 (2.3) |
|  | Cirrhosis | 0 (0.0) | 20 (1.9) | 0 (0.0) | 0 (0.0) | 7 (7.1) | 10 (1.6) | 0 (0.0) |
|  | Neoplasm | 0 (0.0) | 212 (20.7) | 0 (0.0) | 11 (9.4) | 12 (12.2) | 83 (13.6) | 0 (0.0) |
|  | Autoimmune disorders | 0 (0.0) | 2 (0.2) | 0 (0.0) | 14 (12.0) | 2 (2.0) | 47 (7.7) | 0 (0.0) |
| Obesity^a^ |  | N=73 | N=1026 | N=399 | N=117 | N=98 | N=609 | N=386 |
|  | Yes | 3 (4.1) | 219 (21.3) | 2 (0.5) | 27 (23.1) | 30 (30.6) | 41 (6.7) | 0 (0.0) |
| Outpatient consultations last 3 months |  | - | - | N=399 | N=117 | N=95 | N=609 | N=141 |
|  | 0 | - | - | 275 (68.9) | 43 (36.8) | 21 (22.1) | 161 (26.4) | 32 (22.7) |
|  | 1 | - | - | 50 (12.5) | 30 (25.6) | 31 (32.6) | 75 (12.3) | 43 (30.5) |
|  | > 1 | - | - | 74 (18.5) | 44 (37.6) | 43 (45.3) | 373 (61.2) | 66 (46.8) |
| Smoking habits^b^ |  | N=73 | N=854 | N=399 | N=117 | N=98 | N=609 | N=386 |
|  | Never smoker | 29 (39.7) | 378 (44.3) | 173 (43.4) | 60 (51.3) | 43 (43.9) | 332 (54.5) | 375 (97.2) |
|  | Past smoker | 25 (34.2) | 297 (34.8) | 17 (4.3) | 26 (22.2) | 34 (34.7) | 140 (23.0) | 2 (0.5) |
|  | Current smoker | 19 (26.0) | 179 (21.0) | 209 (52.4) | 31 (26.5) | 21 (21.4) | 137 (22.5) | 9 (2.3) |
| Functional status impairment (Barthel Index)^c^ |  | - | N=562 | - | N=54 | N=56 | N=200 | - |
|  | Total (0-15) | - | 10 (1.8) | - | 0 (0.0) | 1 (1.8) | 4 (2.0) | - |
|  | Severe (20-35) | - | 5 (0.9) | - | 2 (3.7) | 1 (1.8) | 3 (1.5) | - |
|  | Moderate (40-55) | - | 17 (3.0) | - | 1 (1.9) | 1 (1.8) | 5 (2.5) | - |
|  | Mild (60-90) | - | 124 (22.1) | - | 33 (61.1) | 20 (35.7) | 35 (17.5) | - |
|  | Minimal (95-100) | - | 354 (63.0) | - | 17 (31.5) | 32 (57.1) | 153 (76.5) | - |
| Influenza vaccination ≥14 days from symptom onset |  | N=73 | N=1026 | N=399 | N=117 | N=98 | N=609 | N=386 |
|  | Yes | 7 (9.6) | 293 (28.6) | 4 (1.0) | 16 (13.7) | 42 (42.9) | 20 (3.3) | 0 (0.0) |
| Antiviral use during the current episode |  | N=73 | N=1026 | B=399 | N=117 | N=98 | N=609 | N=386 |
|  | Yes | 1 (1.4) | 739 (72.0) | 374 (93.7) | 33 (28.2) | 48 (49.0) | 270 (44.3) | 0 (0.0) |

Abbreviation: COPD, chronic obstructive pulmonary disease

^a^ Assessed only in patients aged ≥18 years

^b^ For patients aged <18 years, represents second-hand smoke was assessed

^c^ Measured only for patients aged ≥65 years
