# Supplementary material for: Complicated hospitalization due to influenza: results from the Global Hospital Influenza Network for the 2017–2018 season
Source: BMC Infect Dis. 2020 Jul 2;20:465. doi: 10.1186/s12879-020-05167-4 (PMC7330273; doi:10.1186/s12879-020-05167-4)
Supplement: Supplementary file 6 — Additional file 6: Supplemental Table 6. Characteristics of influenza-positive patients by site (Mexico, Romania, Moscow, St. Petersburg, Serbia, South Africa, and Spain). [file 12879_2020_5167_MOESM6_ESM.docx]

**Supplemental Table 6. Characteristics of influenza-positive patients by site (Mexico, Romania, Moscow, St. Petersburg, Serbia, South Africa, and Spain)**

|  |  |  |  |  | **n (%)** |  |  |  |
| --- | --- | --- | --- | --- | --- | --- | --- | --- |
| **Characteristic** | **Category** | **Mexico** | **Romania** | **Moscow** | **St. Petersburg** | **Serbia** | **South Africa** | **Spain** |
| Age |  | N=701 | N=492 | N=1247 | N=3101 | N=590 | N=1123 | N=2841 |
|  | <1 y | 146 (20.8) | 39 (7.9) | 76 (6.1) | 580 (18.7) | 24 (4.1) | 517 (46.0) | 271 (9.5) |
|  | 1 to <5 y | 239 (34.1) | 182 (37.0) | 328 (26.3) | 983 (31.7) | 19 (3.2) | 231 (20.6) | 223 (7.8) |
|  | 5 to <15 y | 64 (9.1) | 103 (20.9) | 164 (13.2) | 371 (12.0) | 17 (2.9) | 18 (1.6) | 66 (2.3) |
|  | 15 to <50 y | 110 (15.7) | 89 (18.1) | 537 (43.1) | 790 (25.5) | 136 (23.1) | 172 (15.3) | 234 (8.2) |
|  | 50 to <65 y | 51 (7.3) | 50 (10.2) | 69 (5.5) | 199 (6.4) | 164 (27.8) | 119 (10.6) | 361 (12.7) |
|  | 65 to <75 y | 35 (5.0) | 14 (2.8) | 24 (1.9) | 105 (3.4) | 149 (25.3) | 38 (3.4) | 469 (16.5) |
|  | 75 to <85 y | 43 (6.1) | 13 (2.6) | 29 (2.3) | 62 (2.0) | 71 (12.0) | 25 (2.2) | 641 (22.6) |
|  | ≥85 y | 13 (1.9) | 2 (0.4) | 20 (1.6) | 11 (0.4) | 10 (1.7) | 3 (0.3) | 576 (20.3) |
| Sex |  | N=701 | N=492 | N=1247 | N=3101 | N=590 | N=1123 | N=2841 |
|  | Female | 326 (46.5) | 251 (51.0) | 828 (66.4) | 1459 (47.0) | 271 (45.9) | 510 (45.4) | 1338 (47.1) |
|  | Male | 375 (53.5) | 241 (49.0) | 419 (33.6) | 1642 (53.0) | 319 (54.1) | 613 (54.6) | 1503 (52.9) |
| Chronic Conditions |  | N=701 | N=492 | N=1247 | N=3101 | N=590 | N=1123 | N=2841 |
|  | 0 | 401 (57.2) | 386 (78.5) | 1024 (82.1) | 2643 (85.2) | 130 (22.0) | 960 (85.5) | 845 (29.7) |
|  | 1 | 174 (24.8) | 63 (12.8) | 140 (11.2) | 337 (10.9) | 244 (41.4) | 151 (13.4) | 788 (27.7) |
|  | > 1 | 126 (18.0) | 43 (8.7) | 83 (6.7) | 121 (3.9) | 216 (36.6) | 12 (1.1) | 1208 (42.7) |
| Hospitalized within the last 12 months |  | N=701 | N=492 | N=1247 | N=3101 | N=590 | N=1123 | N=2841 |
|  | Yes | 323 (46.1) | 169 (34.3) | 224 (18.0) | 791 (25.5) | 275 (46.6) | 400 (35.6) | 862 (30.3) |
| Underlying chronic conditions |  | N=701 | N=492 | N=1247 | N=3101 | N=590 | N=1123 | N=2841 |
|  | Cardiovascular disease | 128 (18.3) | 53 (10.8) | 133 (10.7) | 280 (9.0) | 224 (38.0) | 9 (0.8) | 1169 (41.1) |
|  | COPD | 70 (10.0) | 10 (2.0) | 25 (2.0) | 89 (2.9) | 166 (28.1) | 5 (0.4) | 769 (27.1) |
|  | Asthma | 85 (12.1) | 9 (1.8) | 35 (2.8) | 67 (2.2) | 26 (4.4) | 27 (2.4) | 235 (8.3) |
|  | Diabetes | 60 (8.6) | 19 (3.9) | 31 (2.5) | 52 (1.7) | 82 (13.9) | 29 (2.6) | 730 (25.7) |
|  | Immunological disorders | 18 (2.6) | 18 (3.7) | 2 (0.2) | 12 (0.4) | 38 (6.4) | 98 (8.7) | 43 (1.5) |
|  | Rheumatological disorders | 23 (3.3) | 8 (1.6) | 8 (0.6) | 14 (0.5) | 4 (0.7) | 2 (0.2) | 0 (0.0) |
|  | Renal disease | 32 (4.6) | 9 (1.8) | 32 (2.6) | 19 (0.6) | 74 (12.5) | 2 (0.2) | 358 (12.6) |
|  | Neuromuscular disorders | 35 (5.0) | 14 (2.8) | 17 (1.4) | 40 (1.3) | 40 (6.8) | 0 (0.0) | 146 (5.1) |
|  | Cirrhosis | 7 (1.0) | 22 (4.5) | 12 (1.0) | 18 (0.6) | 11 (1.9) | 0 (0.0) | 99 (3.5) |
|  | Neoplasm | 17 (2.4) | 6 (1.2) | 29 (2.3) | 10 (0.3) | 70 (11.9) | 2 (0.2) | 217 (7.6) |
|  | Autoimmune disorders | 12 (1.7) | 4 (0.8) | 16 (1.3) | 9 (0.3) | 14 (2.4) | 1 (0.1) | 109 (3.8) |
| Obesity^a^ |  | N=701 | N=492 | N=1247 | N=3101 | N=590 | N=1123 | N=2841 |
|  | Yes | 78 (11.1) | 28 (5.7) | 107 (8.6) | 150 (4.8) | 45 (7.6) | 2 (0.2) | 600 (21.1) |
| Outpatient consultations last 3 months |  | N=701 | N=489 | N=1245 | N=3101 | N=424 | N=941 | N=2816 |
|  | 0 | 121 (17.3) | 239 (48.9) | 742 (59.5) | 1664 (53.7) | 4 (0.9) | 783 (83.2) | 763 (27.1) |
|  | 1 | 260 (37.1) | 99 (20.2) | 195 (15.6) | 912 (29.4) | 34 (8.0) | 105 (11.2) | 353 (12.5) |
|  | > 1 | 320 (45.6) | 151 (30.9) | 308 (24.7) | 525 (16.9) | 386 (91.0) | 53 (5.6) | 1700 (60.4) |
| Smoking habits^b^ |  | N=701 | N=492 | N=1247 | N=3101 | N=590 | N=1123 | N=2841 |
|  | Never smoker | 388 (55.3) | 277 (56.3) | 904 (72.5) | 1757 (56.7) | 205 (34.7) | 854 (76.0) | 1543 (54.3) |
|  | Past smoker | 146 (20.8) | 78 (15.9) | 113 (9.1) | 494 (15.9) | 249 (42.2) | 67 (6.0) | 830 (29.2) |
|  | Current smoker | 167 (23.8) | 137 (27.8) | 230 (18.4) | 850 (27.4) | 136 (23.1) | 202 (18.0) | 468 (16.5) |
| Functional status impairment (Barthel index)^c^ |  | N=91 | N=29 | N=73 | N=162 | N=229 | N=16 | N=1684 |
|  | Total (0-15) | 6 (6.6) | 1 (3.4) | 0 (0.0) | 2 (1.2) | 15 (6.6) | 2 (12.5) | 132 (7.8) |
|  | Severe (20-35) | 4 (4.4) | 0 (0.0) | 0 (0.0) | 0 (0.0) | 44 (19.2) | 1 (6.3) | 69 (4.1) |
|  | Moderate (40-55) | 8 (8.8) | 1 (3.4) | 5 (6.8) | 0 (0.0) | 84 (36.7) | 1 (6.3) | 99 (5.9) |
|  | Mild (60-90) | 20 (22.0) | 26 (89.7) | 39 (53.4) | 44 (27.2) | 86 (37.6) | 3 (18.8) | 402 (23.9) |
|  | Minimal (95-100) | 43 (47.3) | 1 (3.4) | 23 (31.5) | 104 (64.2) | 0 (0.0) | 6 (37.5) | 884 (52.5) |
| Influenza vaccination ≥14 days from symptom onset |  | N=701 | N=492 | N=1247 | N=3101 | N=590 | N=1123 | N=2841 |
|  | Yes | 86 (12.3) | 17 (3.5) | 62 (5.0) | 142 (4.6) | 28 (4.7) | 1 (0.3) | 1100 (38.7) |
| Antiviral use during the current episode |  | N=701 | N=492 | N=1247 | N=3101 | N=490 | N=1123 | N=2841 |
|  | Yes | 44 (6.3) | 445 (90.4) | 33 (2.6) | 219 (7.1) | 0 (0.0) | 4 (0.4) | 334 (11.8) |

Abbreviation: COPD, chronic obstructive pulmonary disease

^a^ Assessed only in patients aged ≥18 years

^b^ For patients aged <18 years, represents second-hand smoke was assessed

^c^ Measured only for patients aged ≥65 years
