# Supplementary material for: Complicated hospitalization due to influenza: results from the Global Hospital Influenza Network for the 2017–2018 season
Source: BMC Infect Dis. 2020 Jul 2;20:465. doi: 10.1186/s12879-020-05167-4 (PMC7330273; doi:10.1186/s12879-020-05167-4)
Supplement: Supplementary file 8 — Additional file 8: Supplemental Figure 2. Description of data: Influenza strain circulation by year-week for individual sites within the South West Europe influenza transmission zone. [file 12879_2020_5167_MOESM8_ESM.docx]

**
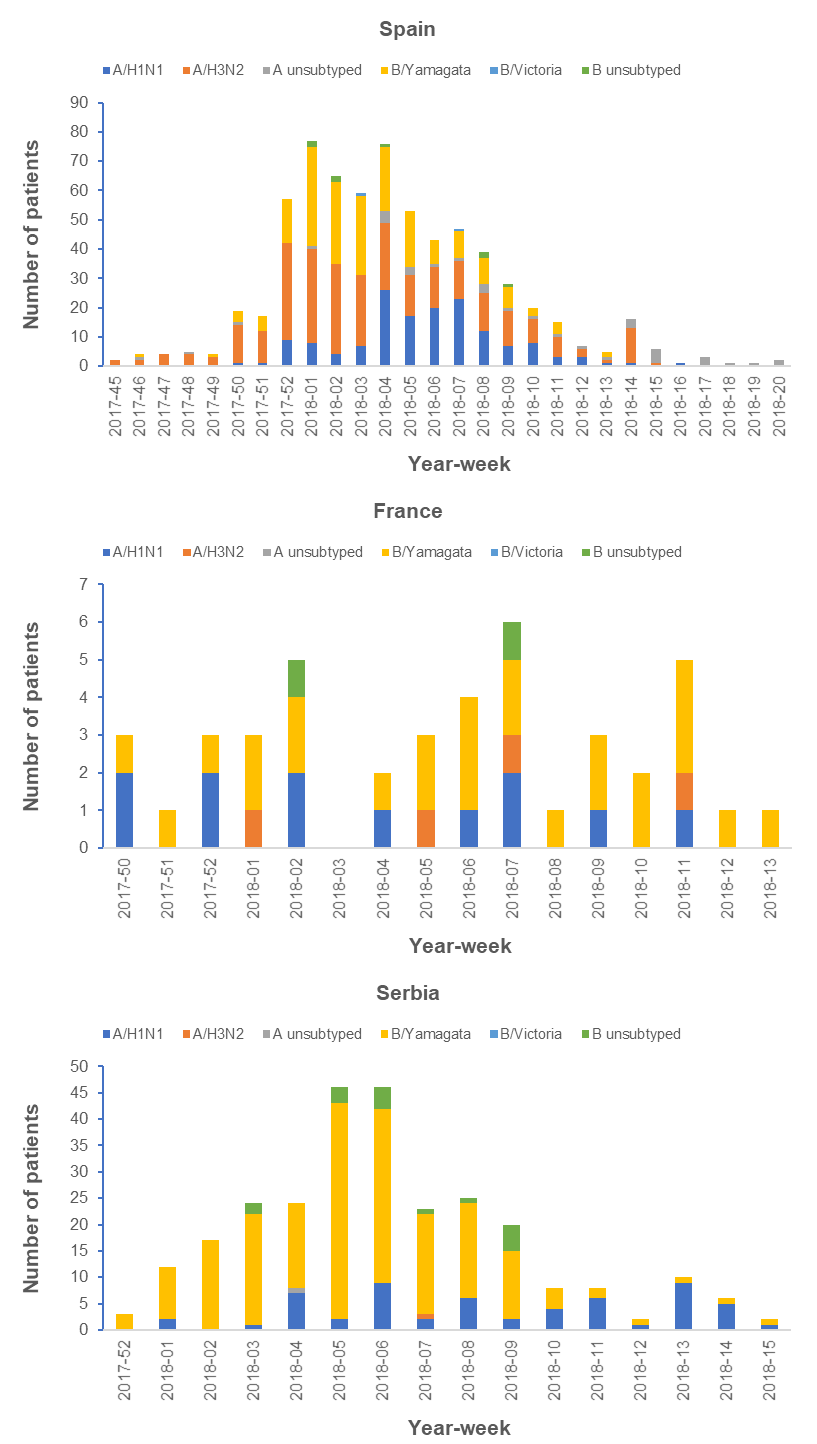
**

**Supplemental Figure 2.** **Influenza strain circulation by year-week for individual sites within the South West Europe influenza transmission zone**

Influenza strains were detected by reverse transcription-polymerase chain reaction. An individual patient could have been positive for more than one strain of influenza.
