# Supplementary material for: Complicated hospitalization due to influenza: results from the Global Hospital Influenza Network for the 2017–2018 season
Source: BMC Infect Dis. 2020 Jul 2;20:465. doi: 10.1186/s12879-020-05167-4 (PMC7330273; doi:10.1186/s12879-020-05167-4)
Supplement: Supplementary file 9 — Additional file 9: Supplemental Figure 3. Proportion of influenza-positive patients admitted to an ICU, requiring mechanical ventilation, or that died while hospitalized by age group. [file 12879_2020_5167_MOESM9_ESM.docx]

**
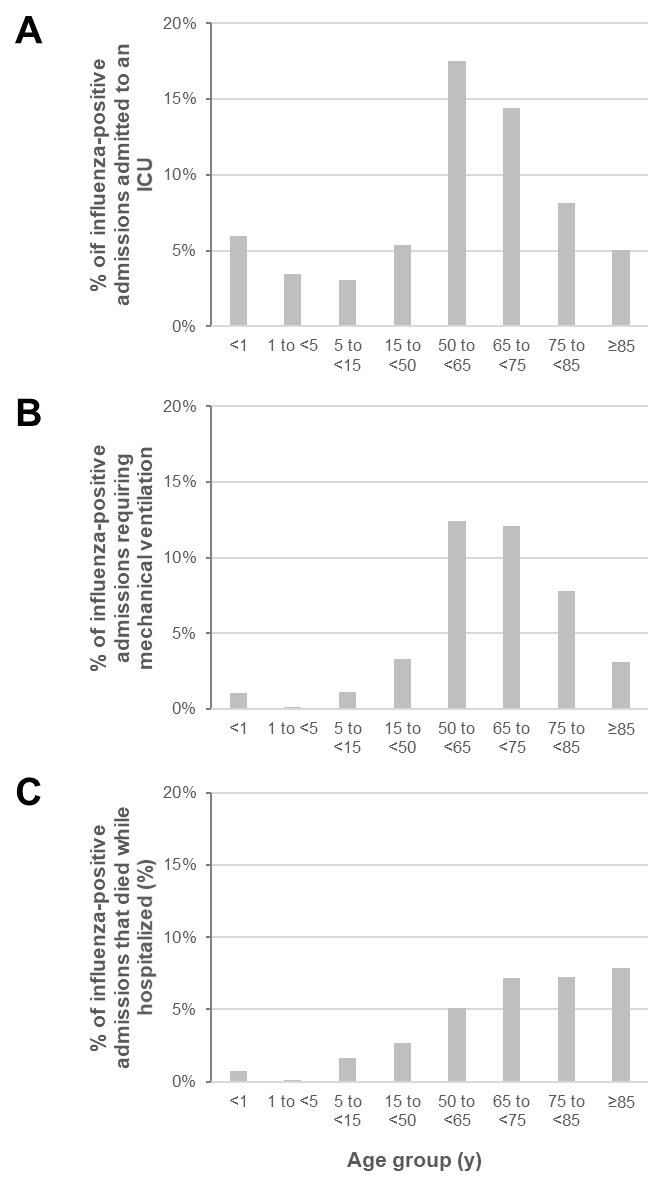
**

**Supplemental Figure 3. Proportion of influenza-positive patients admitted to an ICU (A), requiring mechanical ventilation (B), or that died while hospitalized (C) by age group**
