# Supplementary material for: Complicated hospitalization due to influenza: results from the Global Hospital Influenza Network for the 2017–2018 season
Source: BMC Infect Dis. 2020 Jul 2;20:465. doi: 10.1186/s12879-020-05167-4 (PMC7330273; doi:10.1186/s12879-020-05167-4)
Supplement: Supplementary file 10 — Additional file 10: Supplemental Table 7. Factors associated with ICU admission, mechanical ventilation, and death during hospitalization in influenza-positive patients. [file 12879_2020_5167_MOESM10_ESM.pdf]

Supplemental Table 7. Factors associated with ICU admission, mechanical ventilation, and death during hospitalization in influenza-positive patients

		OR (95% CI)		
**Factor**	**Age group**	**ICU admission**	**Mechanical ventilation**	**Death during hospitalization**
COPD	15 to <50	0.69 (0.10, 4.87)	1.47 (0.12, 18.81)	-
	50 to <65	**2.88 (1.21, 6.84)**	2.17 (0.91, 5.19)	0.60 (0.15, 2.40)
	≥65	**2.84 (1.44, 5.57)**	**2.33 (1.33, 4.07)**	0.89 (0.49, 1.62)
Cardiovascular disease	15 to <50	**4.11 (1.29, 13.13)**	0.74 (0.12, 4.68)	-
	50 to <65	1.73 (0.78, 3.84)	1.08 (0.46, 2.56)	0.54 (0.17, 1.71)
	≥65	1.00 (0.50, 2.01)	1.02 (0.57, 1.81)	**1.88 (1.01, 3.50)**
Diabetes	15 to <50	2.87 (0.85, 9.65)	2.28 (0.50, 10.43)	-
	50 to <65	1.59 (0.60, 4.23)	2.67 (0.97, 7.39)	0.95 (0.22, 4.19)
	≥65	1.08 (0.50, 2.32)	1.28 (0.71, 2.33)	0.96 (0.53, 1.73)
Obesity	15 to <50	2.18 (0.75, 6.36)	3.69 (1.00, 13.63)	-
	50 to <65	0.92 (0.34, 2.50)	0.62 (0.20, 1.91)	1.91 (0.22, 4.19)
	≥65	1.90 (0.75, 4.82)	**2.64 (1.36, 5.13)**	1.15 (0.58, 2.30)
Other chronic conditions	15 to <50	1.26 (0.50, 3.21)	2.29 (0.78, 6.69)	-
	50 to <65	0.52 (0.21, 1.29)	0.55 (0.20, 1.48)	1.46 (0.45, 4.72)
	≥65	0.71 (0.33, 1.52)	0.66 (0.35, 1.25)	1.52 (0.84, 2.76)
Male	<15	1.20 (0.64, 2.26)	1.49 (0.26, 8.63)	1.20 (0.29, 5.07)
	15 to <50	1.21 (0.50, 2.91)	1.30 (0.47, 3.57)	-
	50 to <65	2.20 (0.97, 4.96)	**3.11 (1.28, 7.59)**	**8.34 (2.08, 33.47)**
	≥65	0.83 (0.42, 1.64)	0.65 (0.37, 1.14)	1.18 (0.67, 2.08)
Current smoker	15 to <50	1.76 (0.71, 4.38)	0.53 (0.14, 1.94)	-
	50 to <65	1.20 (0.53, 2.73)	0.79 (0.34, 1.85)	0.38 (0.10, 1.39)
	≥65	3.26 (1.44, 7.35)	2.09 (1.00, 4.39)	0.93 (0.37, 2.33)
Influenza B (vs. A)	<15	0.94 (0.44, 1.99)	**13.44 (1.32, 136.8)**	3.32 (0.69, 16.04)
	15 to <50	0.67 (0.23, 1.92)	0.28 (0.07, 1.22)	-
	50 to <65	0.55 (0.20, 1.49)	0.71 (0.26, 1.99)	0.44 (0.11, 1.75)
	≥65	0.93 (0.40, 2.13)	1.01 (0.52, 1.95)	0.56 (0.29, 1.08)
Use of antivirals during the current influenza episode	<15	0.98 (0.21, 4.53)	None observed	None observed
	15 to <50	**3.86 (1.37, 10.82)**	**3.87 (1.13, 13.28)**	-
	50 to <65	**4.80 (1.53, 15.08)**	1.20 (0.36, 4.01)	0.99 (0.22, 4.38)
	≥65	1.74 (0.60, 5.02)	1.11 (0.53, 2.34)	0.76 (0.39, 1.47)
Hospitalization during the last 12 months	<15	0.90 (0.42, 1.93)	3.78 (0.71, 20.21)	1.20 (0.26, 5.53)
	15 to <50	0.86 (0.33, 2.25)	0.40 (0.11, 1.41)	-
	50 to <65	**2.97 (1.28, 6.91)**	1.86 (0.78, 4.42)	2.86 (0.89, 9.22)
	≥65	0.58 (0.28, 1.21)	0.84 (0.47, 1.51)	1.46 (0.83, 2.56)
Any chronic condition	<15	2.24 (0.88, 5.76)	0.94 (0.07, 11.95)	0.88 (0.06, 13.44)

Abbreviations: ICU, intensive care unit; OR, odds ratio

Adjusted ORs were determined by mixed effects logistic regression adjusted for age and vaccination for influenza during the previous 12 months and with site as a random effect. For patients aged 15 to <65 years (N=1,193) and ≥65 years (N=776), covariates included cardiovascular disease, COPD, diabetes, obesity, other chronic conditions, sex, current smoking, influenza B, antiviral use during the current influenza episode, hospitalization during the previous 12 months, site (as random effect), vaccination, and age. For patients aged <15 years (N=1,309), covariates included any chronic condition, sex, age, influenza B, antiviral use during the current influenza episode, hospitalization during the previous 12 months, site (as random effect), vaccination, and age. Factors associated with ICU admission, mechanical ventilation, or death during hospitalization are indicated in bold text.
